# Self-recovery scheme for audio restoration using auditory masking

**DOI:** 10.1371/journal.pone.0204442

**Published:** 2018-09-28

**Authors:** Alejandra Menendez-Ortiz, Claudia Feregrino-Uribe, Jose Juan Garcia-Hernandez

**Affiliations:** 1 Computer Science Department, INAOE, Luis Enrique Erro #1, Sta. Ma. Tonantzintla, Puebla, CP. 72840, Mexico; 2 Centro de Investigacion y de Estudios Avanzados del IPN, Unidad Tamaulipas, Km. 5.5 Carr. Cd. Victoria-Solo La Marina, Cd. Victoria, Tamps. CP. 87130, Mexico; Nanjing University of Information Science and Technology, CHINA

## Abstract

Self-recovery schemes identify and restore tampering, using as a reference a compressed representation of a signal embedded into itself. In addition, audio self-recovery must comply with a transparency threshold, adequate for applications such as on-line music distribution or speech transmission. In this manuscript, an audio self-recovery scheme is proposed. Auditory masking properties of the signals are used to determine the frequencies that better mask the embedding distortion. Frequencies in the Fourier domain are mapped to the intDCT domain for embedding and extraction of reference bits for signal restoration. The contribution of this work is the use of auditory masking properties for the frequency selection and the mapping to the intDCT domain. Experimental results demonstrate that the proposed scheme satisfies a threshold of -2 ODG, suitable for audio applications. The efficacy of the scheme, in terms of its restoration capabilities, is also shown.

## Introduction

Technologies that allow the sharing and modification of digital content have arisen rapidly in recent years. Many of these technologies facilitate the modification of digital images, videos, and audio. However, there are cases where the owners do not wish unauthorized modifications of their content. Fragile watermarking was devised as a means to authenticate digital content and, in some applications, for tamper localization [1, 2]. Once the schemes were capable of identifying the positions where tampering had occurred, a natural desire was to restore the tampered regions and with this idea self-recovery schemes arose. The scheme proposed by [3] was the first to introduce the idea of self-embedding the image to restore the tampered regions.

To date, many schemes designed for images have been proposed, some of them such as [4, 5], deal with images in the spatial domain and focus on resisting content replacement attacks. Others, such as the schemes proposed by [6–8], deal with signal processing or cropping attacks. A few self-recovery schemes for video signals have also been proposed by [9–12]. There are self-recovery schemes for images that even achieve perfect restoration of the tampered content, provided that the attacked areas are small [13–15].

There are schemes for audio signals that can authenticate and determine whether tampering occurred, such as [16–20]. Self-recovery schemes for speech, such as in [21, 22], have been proposed and can obtain an approximate restoration of the speech signals. A self-recovery scheme for audio signals that reports perfect restoration has been proposed by [23]; however, the results reported are inconclusive. A discussion of these schemes is presented below.

### Application scenarios for audio self-recovery

The first scenario where a self-recovery scheme for audio is required is one for speech restoration. Suppose there is a recorded phone conversation, with this recording subsequently modified to incriminate one of the interlocutors by modifying certain words of his or her speech. This tampered recording could be used against the person; the tampered speech could be submitted to forensic analysis to determine its authenticity.

**Fig 1:**
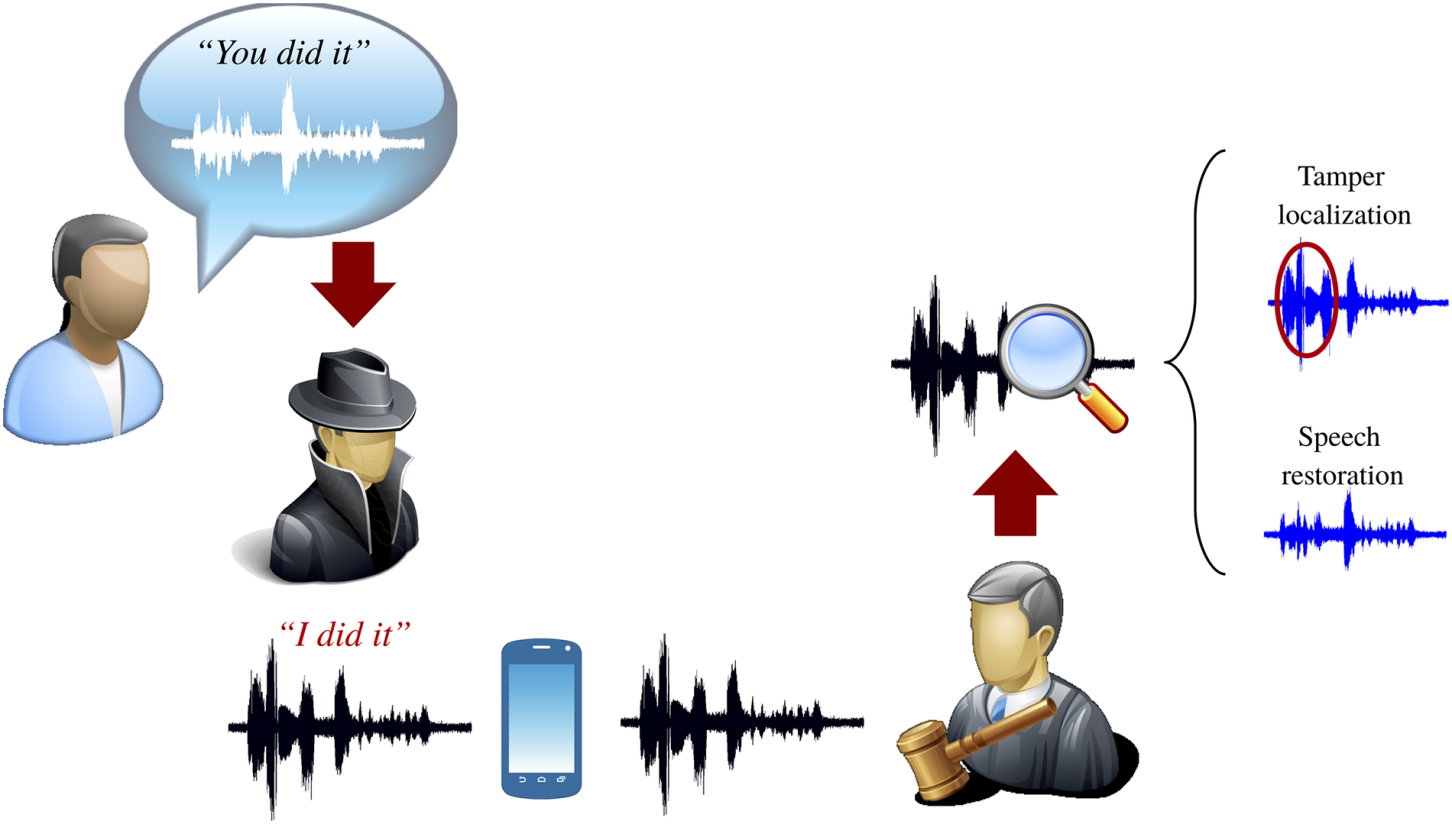
Example of a speech restoration scenario.

A means to obtain the original words from the tampered speech could be part of the repair process of audio forensics [24], and could prove the innocence of the accused. An example of this scenario is presented in Fig 1. A self-recovery scheme for audio is a mechanism that can be used in such a way and that allows the restoration of the original contents of a speech signal.

**Fig 2:**
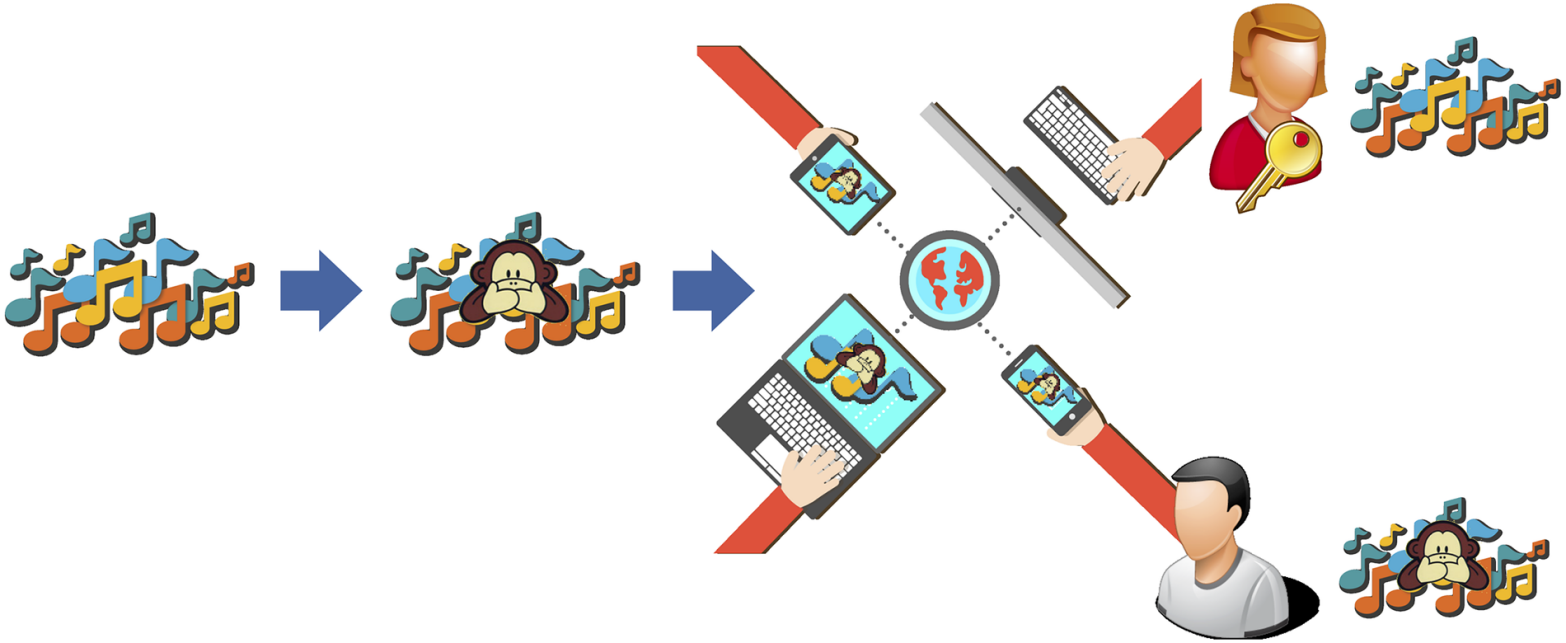
Example of censored music distribution.

The second scenario for audio self-recovery is in the music industry. There are songs that contain inappropriate language; for these songs to be included in radio airplay, the inappropriate content has to be censored by editing the songs. Offending content is removed through re-sampling, bleeping, and replacing words with silence, sound effects, or single tones [25]. In a music distribution scenario, censored songs could be freely distributed, but premium users could pay a fee to remove the censorship. An example of this case is presented in Fig 2.

A self-recovery scheme for audio could be used in this scenario, where the premium users can purchase a key to restore the original contents of the song. Both application scenarios consider the same kind of modification of the audio signals, which is the substitution of regions of the signal by another content. The new content can be taken from another audio signal, or it could be artificially generated, such as the single tone generation for music censorship. In the present paper, these modifications are addressed as content replacement attacks. A content replacement attack consists of substituting a set of samples from an audio signal with another set of samples of the same size.

### Related work

The work presented by [21] proposes a self-recovery scheme for speech signals. It calculates a lossy compressed version of the speech, which is later encoded with Reed—Solomon (RS) codes, which correct errors if tampering occurs. Hash bits are calculated from the MSB of the samples and inserted into the segments for tamper detection. The encoded symbols from the RS codes are permuted, based on a secret key, to secure the information. The permuted symbols, along with the hash bits, are inserted into the two least significant bits (LSB) of the samples in each frame. Although the scheme has low distortion for the payload inserted (16 kbps), due to the LSB substitution strategy used for embedding, the LSB substitution compromises the practicality of the scheme since the watermark becomes very fragile. For instance, a simple change of volume in the signal prevents the detection of the watermark. Furthermore, the watermark can be very easily obtained by an unauthorized user by simply reading the LSB of each sample in the signal. Another disadvantage of this method is that only approximate restoration is possible after the signals have been tampered, since the RS codes are calculated from a lossy compressed speech.

The scheme proposed by [22] is a self-recovery scheme for speech signals. It obtains a compressed version of the speech by calculating a 3-level DWT and a DCT; the coefficients from both transforms are concatenated to form the compressed signal. The speech is divided into segments, and an index for each segment is calculated based on a chaotic map; these indices are embedded at the beginning of each segment to identify tampering. The compressed speech is divided into segments and scrambled prior to the embedding, so the information needed to restore a tampered segment can be extracted from another unaltered segment. The segment indices and the compressed speech are embedded using a quantization strategy. Because the compression strategy is a lossy one, and the quantization used for the embedding is lossy as well, only approximate restoration can be achieved. The speech dataset used to obtain the experimental results is one recorded by the authors. Results obtained with standard datasets are not reported.

[23] introduces a self-recovery scheme for audio signals with perfect restoration capabilities; nonetheless, the experimental results reported do not provide evidence for this claim. This scheme is based on the self-recovery scheme for images by [13]; in the implementation for audio signals, the authors use an Efficient Generalized Integer Transform (EGIT) to insert the check bits and reference bits required for the tamper localization and signal restoration, respectively. The EGIT is an integer mapping of samples that includes a difference expansion strategy to insert watermark bits. An adjustment of the dynamic range of the signals is performed to avoid the construction and insertion of a location map that allows the exact restoration of the original sample values. The experimental results only present the waveforms of one audio signal, where the watermarked, attacked, and restored signals are illustrated; however, the quality of the results for a larger number of signals is not reported, such as ODG, SNR or a similar audio quality metric. Moreover, the dataset or datasets used to evaluate the scheme are not reported. An experiment for 100 audio signals is mentioned, where the signal quality is sacrificed in order to improve the restoration capabilities; however, the SNR reported for the 100 signals is lower than 10 dB, which is not an acceptable audio quality for practical applications, which require at least 35 dB [26]. Experimental results are needed to better evaluate the restoration capabilities, transparency, and payload trade-offs of the scheme.

As can be seen, some efforts have been made to design self-recovery schemes for audio and speech signals. Two self-recovery schemes for speech achieve approximate restoration of the signals after they have been tampered, and they also have an adequate transparency for the watermarked signals; however, the robustness and security of these methods is an issue for practical applications. A self-recovery scheme for audio signals has also been proposed; nonetheless, the experimental results reported are inconclusive and perfect restoration has not been sustained, at least with a transparency of the watermarked signals adequate for some application scenarios. It can be observed that self-recovery schemes for audio and speech signals with perfect restoration is an open problem in the state of the art.

In the present paper, a self-recovery scheme for audio signals that uses auditory masking is introduced: it maintains a transparency within an acceptable threshold for audio applications by exploiting the auditory masking properties of the signal for watermark embedding; a patent describing this scheme has been applied for [27]. This scheme differs from a previous approach by the authors [28] in its restoration capabilities, where the former achieves perfect restoration. It also differs in the selection of frequencies for embedding, the masking threshold strategy allows the reduction of the perceptual impact. Another difference is the modification of the prediction error expansion (PEE) strategy used for watermark embedding: in the present paper, a multi-bit strategy is explored to double the reference bits that can be embedded, thus improving the restoration capabilities. Applications where a self-recovery scheme for audio signals is necessary will be presented forthwith.

The rest of the manuscript is organized as follows. First, the details of the the proposed self-recovery scheme will be presented. Then, the experimental results and a comparison with related work will be given. A discussion of the limitations of the scheme will be given. Finally, the conclusions of the paper and lines for future research will be presented.

## Proposed self-recovery scheme for audio

Self-recovery watermarking schemes originally arose for images with the idea of restoring the missing areas in addition to simply identifying the tampered regions. Although each scheme uses a different strategy, the general ideas for the encoding and decoding processes are as follows. The encoding process calculates reference bits and check bits from the signal. The reference bits are a reduced version of the media itself (calculated by compressing or obtaining a descriptive representation), and the check bits are the result of feeding regions of the signal to a hash function. Both the reference bits and check bits are scattered for embedding, obtaining in this manner the watermarked signal. The decoding process receives a signal and extracts a watermark, from which the extracted reference bits and check bits are obtained. The extracted check bits are compared against the check bits calculated from the received signal to identify the tampered regions. By using the reference bits from non-tampered regions, the tampered reference bits can be restored, and with both the non-tampered and restored reference bits, the tampered areas of the signal can be recovered.

One of the greatest challenges with self-recovery for audio is the distortion caused by the embedding process. The target applications where this scheme is to be used require audio signals with a transparency over -2 ODG. The objective difference grade (ODG) is the transparency metric recommended by ITU-R B.S.1387 [29]. Because of this transparency restriction, a strategy to reduce perceptual impact had to be devised by the use of the integer Discrete Cosine Transform (intDCT) domain for the embedding and extraction of the watermark. The intDCT domain was selected because it gives a representation of the signal in the frequency domain, where the watermark can be inserted selectively in frequency components that better mask the insertion noise. The intDCT also maps an integer time-domain signal to its integer frequency components; these components need to be integers because the embedding and extraction of the watermark is performed with reversible algorithms that require integer values to maintain reversibility.

### intDCT transform

The intDCT domain is used both for the embedding and extraction of the watermark. The forward DCT-IV transform of an *N*-point audio signal *x*[*n*] is given by Eq (1), and its inverse transform is given by Eq (3):

$$\begin{array}{cc} X[m] & =C_{N}^{IV}\cdot x\left[n\right],\\ m=n & =0,1,\cdots ,N-1 \end{array}$$ (1)

where **X** represents the intDCT coefficients of **x**. and $C_{N}^{IV}$ is the transform matrix, defined by

$$C_{N}^{IV}=\sqrt{\frac{2}{N}}[\text{cos}(\frac{\left(m+\frac{1}{2}\right)\left(n+\frac{1}{2}\right)\pi }{N})],$$ (2)

where *m* = 0, 1, ⋯, *N* − 1 and *n* = 0, 1, ⋯, *N* − 1. Because $C_{N}^{IV}$ is an orthogonal matrix, the inverse intDCT transform is given by

$$x\left[n\right]=C_{N}^{IV}\cdot X\left[m\right].$$ (3)

As was already mentioned, the intDCT is used in this work because the embedding and extraction algorithms require an integer representation of the frequency components of the signal. In this implementation, the fast intMDCT algorithm proposed by [30] is used to calculate the intDCT, which is an approximation of the DCT-IV. The fast intMDCT divides the transform matrix into five submatrices; the multiplication by each of these five submatrices is done through a lifting stage with a rounding operation. The intDCT coefficients are obtained through the five lifting stages.

### Encoding process

**Fig 3:**
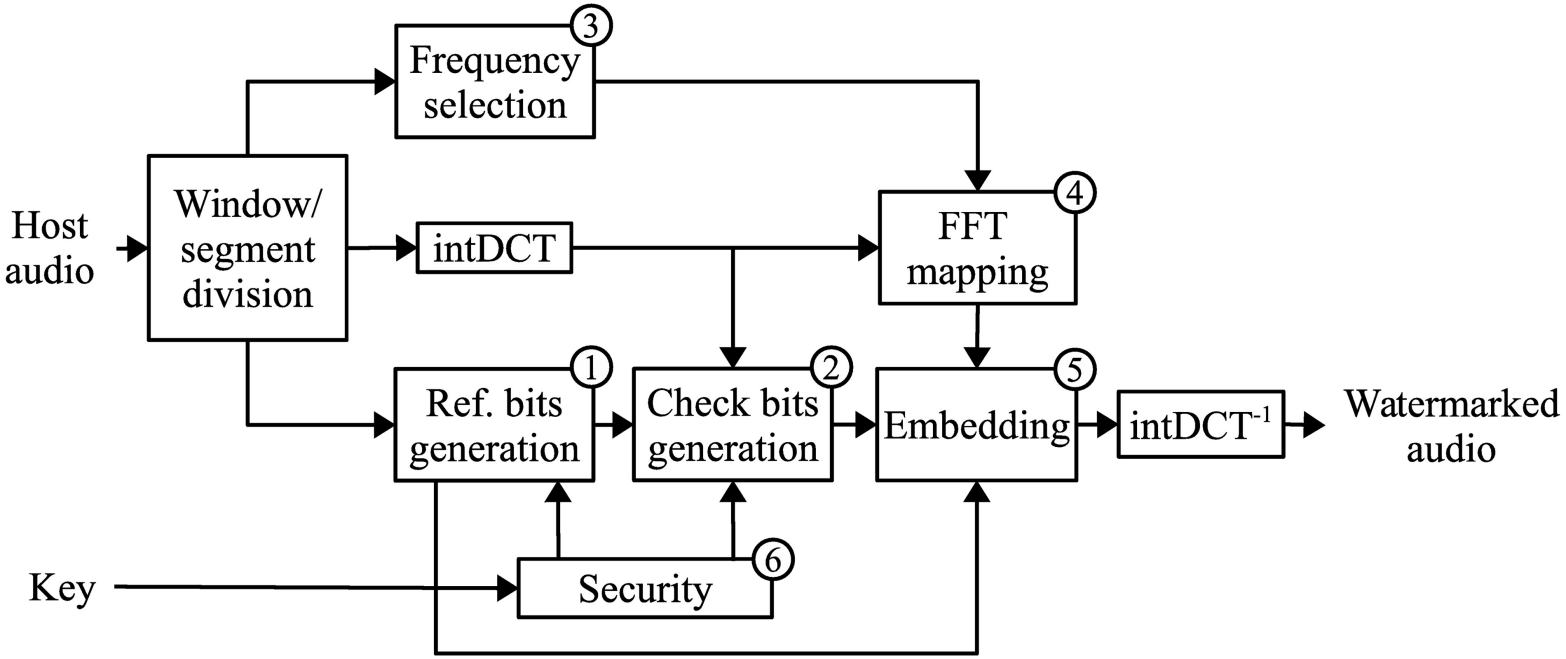
Block diagram of the encoding process.

The general steps of the encoding process in the proposed scheme are presented in Fig 3. Because of the dimensionality of audio signals, it is difficult to process them as a whole, as is done in schemes for images. The proposed self-recovery strategy processes windows of samples. For an audio signal of size *L*, select a window of samples with length *L*_*w*_. There are ⌊*L*/*L*_*w*_⌋ windows for the signal. To increase the accuracy of the tamper detection, and for implementation purposes, each window being processed is divided into segments of length *L*_*s*_, for each window, there are ⌊*L*_*w*_/*L*_*s*_⌋ segments in all. The implementation of the proposed scheme uses windows of size *L*_*w*_ = 44,032 and segments of size *L*_*s*_ = 512.

#### Reference bit generation

In this step, the bits that will be used to restore the signal are generated. The audio signals considered for the scheme have CD quality, where each sample is represented by 16 bits. Since this amount of information cannot be embedded within the signal, it must be reduced. The binary representation for each sample in a window is obtained, producing 16 × *N*_*w*_ bits per window. Pseudo-randomly permute those bits based on the secret key, and reshape them into *N*_*w*_/*n*_*g*_ bit-groups. The variable *n*_*g*_ can take any value that is a power of two and is smaller than the length of the window, *i.e*., *n*_*g*_ = {2^*g*^|2^*g*^ < *N*_*w*_}, where *g* = {1, 2, ⋯}. Each bit-group contains *n*_*b*_ = *n*_*g*_ × 16 bits. Denote the bits in a bit-group by **b**_**t**_[1], **b**_**t**_[2], ⋯, **b**_**t**_[*n*_*b*_] where *t* = 1, 2, ⋯, *N*_*w*_/*n*_*g*_. For each bit-group, calculate *n*_*rb*_ = *n*_*b*_/(16 × cr) reference bits **r**_**t**_[1], **r**_**t**_[2], ⋯, **r**_**t**_[*n*_*rb*_] where ‘cr’ is the compression ratio of the bits, *i.e*., cr = 2 will keep $\frac{1}{32}$ of the 16 × *N*_*w*_ original bits, cr = 4 will keep $\frac{1}{64}$ of the 16 × *N*_*w*_ original bits, and so on. The reference bits are calculated as follows:

$$\begin{array}{cc} \left[\begin{array}{c} r_{t}\left[1\right]\\ r_{t}\left[2\right]\\ \vdots \\ r_{t}\left[n_{rb}\right] \end{array}\right] & =A_{t}\cdot [\begin{array}{c} b_{t}\left[1\right]\\ b_{t}\left[2\right]\\ \vdots \\ b_{t}\left[n_{b}\right] \end{array}],\\ t & =1,2,\cdots ,\frac{N_{w}}{n_{g}}, \end{array}$$ (4)

where *A*_*t*_ are pseudo-random binary matrices of size *n*_*rb*_ × *n*_*b*_, the matrices *A*_*t*_ are calculated based on the secret key, and the arithmetic in Eq (4) is modulo-2. The final reference bits are pseudo-randomly permuted based on the secret key.

These steps are described in Algorithm 1, where the function binaryRep(.) translates each scalar within a vector to 16 scalars that correspond to its binary representation, *i.e*., the sample value {255} is translated to {0, 0, 0, 0, 0, 0, 0, 0, 1, 1, 1, 1, 1, 1, 1, 1}. The function randPermut(.) generates a pseudo-random permutation of a given length, taking the value of ‘key’ as its seed. For this implementation, *N*_*w*_ = 44, 032 was selected to process windows of approximately one second, *n*_*g*_ = 256 was determined experimentally to divide the binary representation of the signal for an adequate dispersion of the reference bits. With these values, 44, 032/256 = 172 bit-groups are constructed, and each bit-group contains *n*_*b*_ = 4, 096 bits. The compression ratio is set to two, which means that there are *n*_*rb*_ = 128 reference bits per group. The *A*_*t*_ matrices have sizes of 128 × 4,096, and a total of *N*_*w*_/2 = 22, 016 reference bits are obtained.

**Algorithm 1**: Reference bits generation.

**Input**: Time-domain audio (**x**), window size (*N*_*w*_), number of groups (*n*_*g*_), compression ratio (*c*_*r*_), secret key (key)

**Output**: Reference bits (**r**_**t**_)

**1** **x**_bin_ ← binaryRep(**x**)

**2** perm ← randPermut(|**x**_bin_|, key)

**3** **x**_perm_ ← **x**_bin_(perm)

**4** *n*_*b*_ ← *n*_*g*_ × 16

**5** *t* ← ⌊*N*_*w*_/*n*_*g*_⌋

**6** **for** *n* = 1: *t* **do**     /* Divide into groups */

**7**  **b**_**t**_(:, *n*) ← **x**_perm_((*n* − 1) × *n*_*b*_ + 1: *n* × *n*_*b*_)

**8** **end**

**9** *n*_*rb*_ ← *n*_*b*_/(16 × *c*_*r*_)

**10** *A* ← randi([0 1], [*n*_*rb*_, *n*_*b*_, *t*])

**11** **for** *n* = 1: *t* **do**     /* Calculate reference bits */

**12**  *A*_*t*_ ← *A*(:,:, *n*)

**13**  **r**_**t**_(:, *n*) ← mod(*A*_*t*_ × **b**_**t**_(:, *n*), 2)

**14** **end**

**15** perm ← randPermut(⌊*N*_*w*_/*c*_*r*_⌋, key)

**16** **r**_**t**_ ← reshape(**r**_**t**_, 1, [])

**17** **r**_**t**_ ← **r**_**t**_(perm)

#### Check bit generation

**Fig 4:**
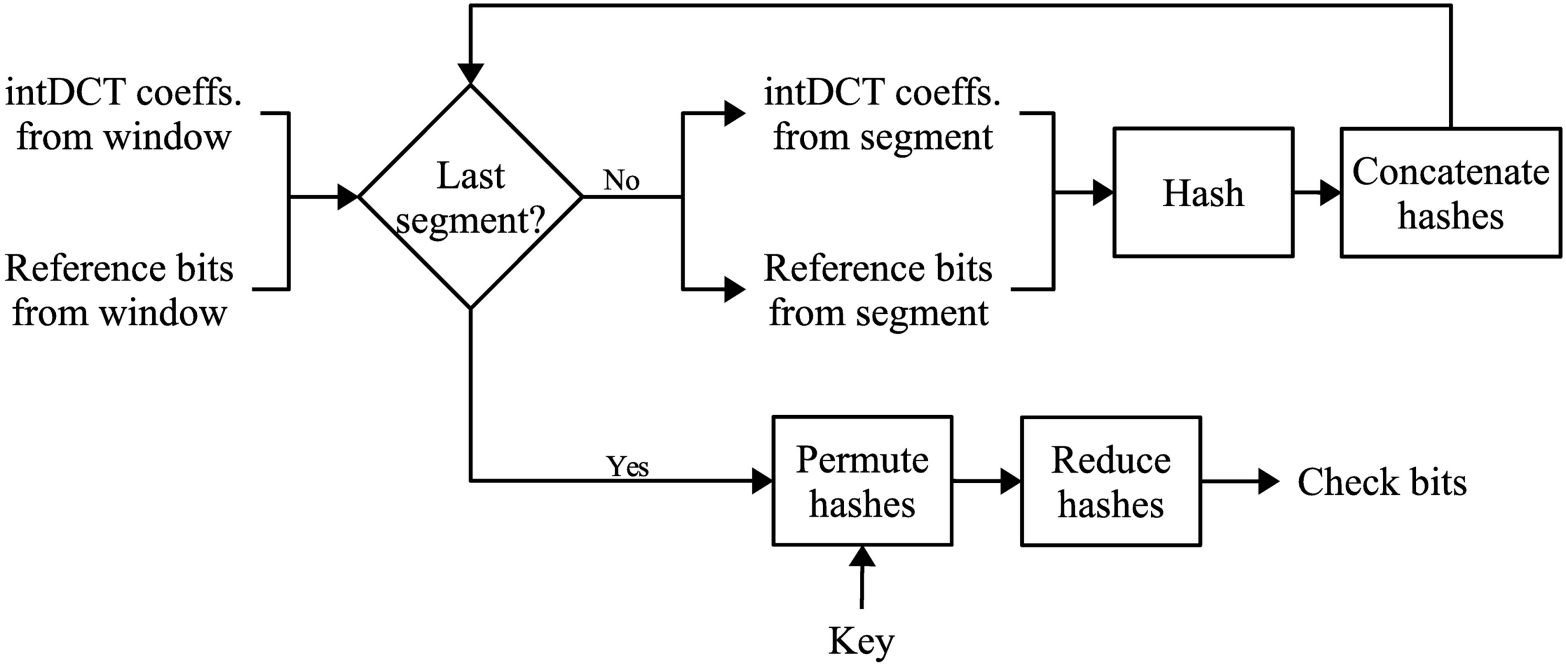
Block diagram for check bits generation.

This step calculates the check bits that will be used to identify the segments of the signal where tampering occurs. Because any modification in the intDCT domain affects all the time domain samples in a segment of audio, there is no way of knowing, just from the time-domain representation of a signal, which samples carry watermark information and which samples do not. For this reason, the check bits are obtained from the intDCT coefficients. For each segment in the window, calculate its forward intDCT transform. Collect the intDCT coefficients, and the reference bits that correspond to the segment. Feed these values to a hash function that produces 256 hash bits per segment. In all, there are $256\times \frac{L_{w}}{L_{s}}$ hash bits per window. Pseudo-randomly permute the hash bits from the whole window, using the secret key to determine the order. To reduce the number of check bits, divide the hash bits into *L*_*w*_/4 subsets, then calculate a modulo-2 sum of the four hash bits in each subset; the sum will produce 64 check bits per segment and $64\times \frac{L_{w}}{L_{s}}$ check bits per window. A block diagram that indicates the steps to generate the check bits is presented in Fig 4.

#### Frequency selection

**Fig 5:**
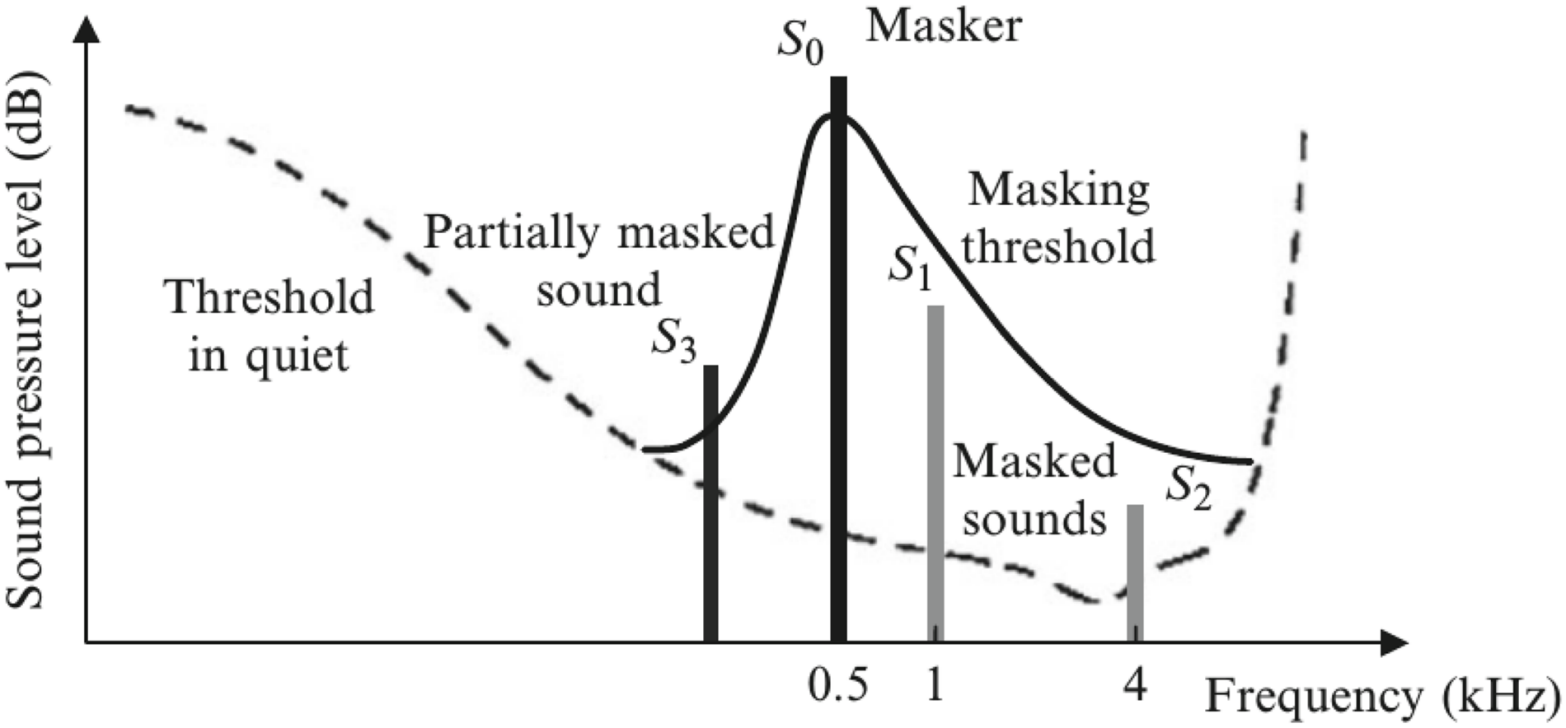
Masking threshold in auditory masking [31].

The use of the intDCT domain is proposed to exploit the selection of frequencies that better mask the noise produced by the insertion of the watermark. This selection is based on the auditory masking in each segment of the signal. Auditory masking occurs when one faint but audible sound (masked sound) is made inaudible in the presence of a louder audible sound (masker) [31]. To determine which frequencies are masked by a predominant frequency, the masking threshold has to be obtained (See Fig 5). The masking threshold indicates the frequency components that are unnoticeable for a human listener because of the existence of a predominant frequency. The predominant frequency ‘masks’ other frequencies near it, therefore, the insertion of a watermark can be done in the masked frequencies without noticeable differences for the human listener. The masking threshold is calculated from the Fourier spectrum of the signal; all the frequencies in the Fourier spectrum that fall under the masking threshold are candidates for embedding.

#### FFT mapping

The FFT spectrum of an *N*-point signal has *N*/2 frequency components, each corresponding to basis functions that linearly increase in frequency. The intDCT of the same signal yields *N* transform coefficients that correspond to cosine basis functions that also linearly increase in frequency; but, unlike the FFT basis functions, the number of periods in each basis function increases in steps of 1/2 [32]. This implies that if the frequency *f*_*i*_ is at the *i*th point in the FFT spectrum, then *f*_*i*_ corresponds to the 2*i*th point in the intDCT domain. Suppose a watermark of length *K* is to be embedded. Select the *K* highest candidate FFT frequencies at indices {*i*_1_, *i*_2_, ⋯, *i*_*K*_}, the corresponding DCT frequencies are at indices {2 × *i*_1_, 2 × *i*_2_, ⋯, 2 × *i*_*K*_}. For natural audio signals, it is expected that the highest frequencies fall under the masking threshold for most of the audio segments. Once the FFT frequencies have been selected as candidates, they are mapped to the intDCT domain for the actual embedding. Because of the mapping from the FFT to the intDCT spectrum previously explained, the intDCT frequencies where the embedding actually occurs are located at even positions. For example, suppose the candidate frequencies in the FFT spectrum are at positions {⋯, 253, 254, 255, 256}. When mapped to the intDCT domain, the positions of these frequencies are {⋯, 506, 508, 510, 512}. As can be seen, frequencies at odd positions were not mapped, because they do not directly correspond to the FFT frequencies due to the increments by 1/2 periods in the intDCT domain. Frequencies at odd positions, *i.e*., frequencies with 1/2 periods, are closely related to the mapped frequencies and since they are high frequencies, it is expected that if frequencies at both even and odd positions are used for embedding, the embedding distortion will remain unnoticeable. In this way, if *M* = *K*/2 frequencies are needed to insert *K* bits (since 2 bits per frequency are inserted), then *M*/2 frequencies in the FFT spectrum that fall under the masking threshold are selected as candidates, their corresponding intDCT frequencies at even positions are mapped and *M*/2 intDCT frequencies in between, *i.e*., the odd positions, are also selected to finally modify the *M* frequencies to insert *K* watermark bits.

#### Embedding

In this final step of the encoding process, the watermark bits to be embedded in each segment are obtained from the reference bits and the check bits previously generated. The watermark bits for each segment are obtained by concatenating *L*_*s*_/2 reference bits with the corresponding 64 check bits of the segment to produce the watermark, denoted by **w**[*k*], where *k* = {1, 2, ⋯, *K*}, and *K* is the size of the watermark. The insertion of the watermark is done through prediction-error expansion (PEE) in the intDCT domain, in a similar fashion to that of the scheme of [33]. It is assumed that coefficients in odd positions are more similar to other coefficients in odd positions, and coefficients in even positions are more similar to other coefficients in even positions, as highlighted in [33]. As mentioned in Section, the intDCT is obtained by multiplying a square matrix by a column vector that corresponds to the audio signal. From the transform matrix $C_{N}^{IV}$ obtained with Eq 2, it can be observed that the absolute sum of the positive values at odd rows is greater than the absolute sum of the negatives, and the absolute sum of the negative values at even rows is greater than that of the positives. The audio signals processed with the scheme are positive integer-valued ones, and it is also assumed that for most natural audio signals, the samples in segments of size 512 have very similar values. The multiplication of the transform matrix C by the integer-valued audio segments results in positive intDCT coefficients at odd positions and negative coefficients at even positions. This holds for most of the segments in all the audio signals tested; therefore, the embedding strategy is based on this assumption. The prediction value of the *i*^th^ coefficient, denoted by $\hat{X}\left[i\right]$ is calculated as

$$\hat{X}\left[i\right]=\lfloor \frac{X[i-2]+X[i-4]}{2}\rfloor ,$$ (5)

and the prediction-error, denoted as *p*, is given by

$$p=X\left[i\right]-\hat{X}\left[i\right],$$ (6)

where *i* represents the index of the mapped intDCT frequency. Two bits are embedded per frequency, and the prediction error *p* is expanded as follows:

$$p_{w}=4\times p+\left(2\times w\left[k\right]\right)+w\left[k+1\right].$$ (7)

The watermarked intDCT coefficients are obtained by

$$Y\left[i\right]=\hat{X}\left[i\right]+p_{w}.$$ (8)

#### Security layer

In a speech restoration scenario such as the one described in Section, the framing person could be interested in rendering it impossible to restore the original speech from the tampered speech. In the music censorship scenario, a customer could desire to restore the original uncensored version of the song without paying the corresponding fee. Both of these things can be done if the secret key used to disperse the reference and check bits can be predicted. If a small key-space is used, a brute force algorithm could find the key. With this key, the reference bits that correspond to a certain region of the speech signal can be found in the rest of the signal; by eliminating those reference bits, the original speech could not be restored. In the other scenario, if the secret key is predicted, a customer can restore the uncensored song without payment of the fee. Because of this, a big enough key-space is necessary. A key such as the one used by the Advanced Encryption Standard (AES) is recommended, *i.e*., a symmetric key of 256 bits.

### Decoding process

**Fig 6:**
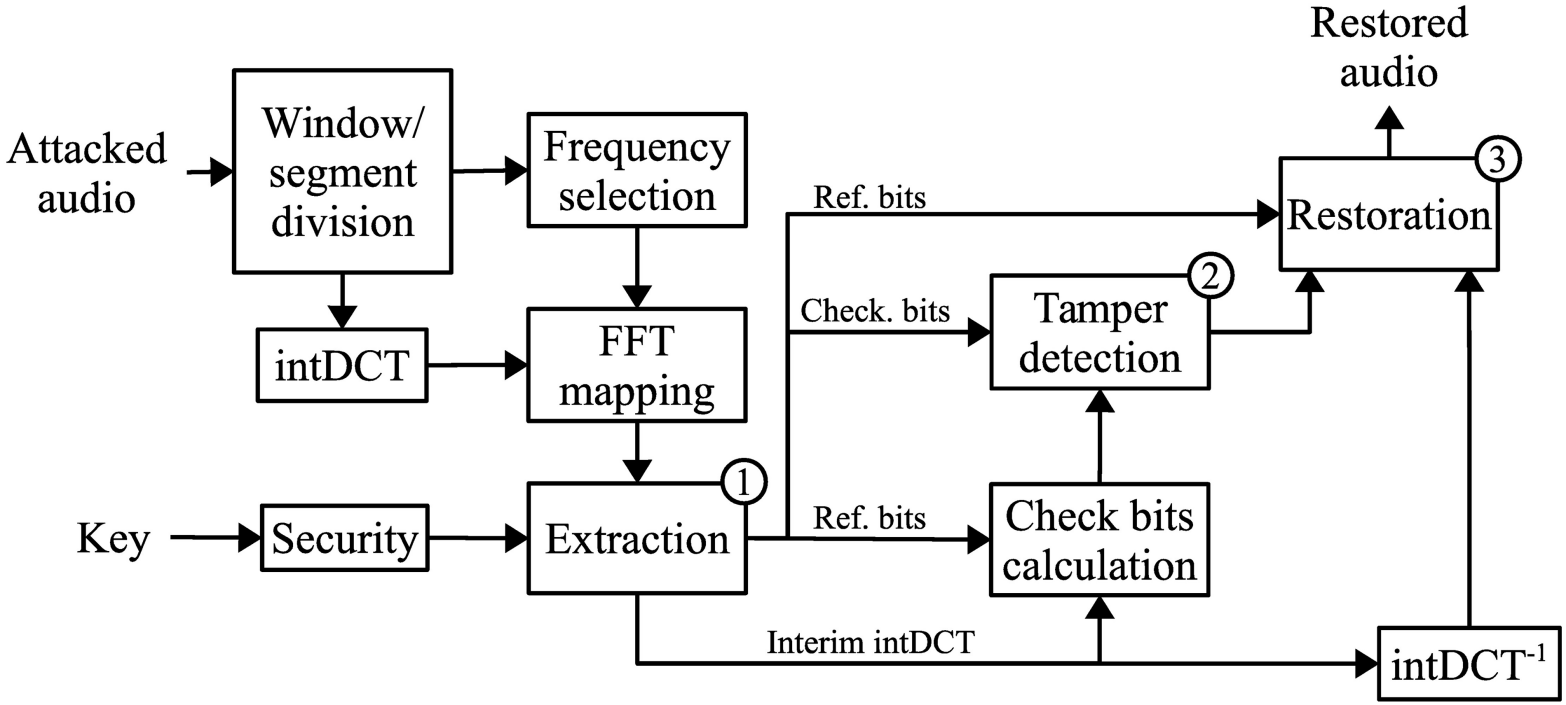
Block diagram of the decoding process.

The steps in the decoding process for the proposed scheme can be seen in Fig 6. As in the encoding process, an audio signal of size *L* is divided into windows of samples of length *L*_*w*_, and each window is further divided into segments of size *Ls*. The decoding process is applied to each of the *L*/*L*_*w*_ windows, and the general steps are detailed below. The watermark is extracted from the intDCT coefficients of each segment. After this extraction has been carried out from all the segments, the reference bits and check bits of the window can be reconstructed using the secret key. The intDCT coefficients are selected using the same masking threshold criteria and FFT mapping used for the embedding. The extracted check bits are compared against the check bits obtained from the received signal to detect the tampered regions. The reference bits and the sample values from the non-tampered regions are used to restore the tampered samples.

#### Watermark extraction

First, each window of signal samples is divided into segments of length *L*_*s*_ as in the encoding process. Then the intDCT coefficients of each segment are selected using the same criteria as in the embedding process. The masking threshold of each segment is obtained, the frequencies in the Fourier spectrum are mapped to the frequencies in the intDCT domain, and the PEE extraction process is applied as follows. The prediction value $\hat{Y}\left[i\right]$ is calculated as

$$\hat{Y}\left[i\right]=\lfloor \frac{Y[i-2]+Y[i-4]}{2}\rfloor ,$$ (9)

and the expanded prediction-error is given by

$$p_{w}=Y\left[i\right]-\hat{Y}\left[i\right],$$ (10)

where *i* represents the indices of the frequencies in the intDCT domain. The original prediction error *p* is obtained by

$$p=\lfloor \frac{p_{w}}{4}\rfloor ,$$ (11)

and the watermark word, *w*_*o*_, which contains two bits, is extracted by

$$\begin{array}{cc} w_{o} & =p_{w}-4\times p,\\ w[k] & =\text{mod}(w_{o},2),\\ w[k+1] & =\text{mod}(\left\lfloor w_{o}/2\right\rfloor ,2). \end{array}$$ (12)

The original intDCT coefficients are restored by

$$X\left[i\right]=\hat{Y}\left[i\right]+p.$$ (13)

The original sample values in the time domain are obtained by applying the inverse intDCT transform to the restored intDCT coefficients. The watermark extracted from each segment is divided into reference bits and check bits. All the reference and check bits of the window are obtained when the watermarks of all the segments have been extracted.

#### Tampered segment identification

The check bits extracted in the previous step are compared against the check bits calculated from the extracted reference bits and the restored sample values from the previous step. The consistency between these check bits is the criterion for judging a segment as *non-tampered* or *tampered*.

To calculate the check bits from the received signal, the non-modified intDCT coefficients of each segment are collected, along with the reference bits that correspond to that segment. All these values are fed to the same hash function, to obtain 256 hash bits per segment. Then the $256\times \frac{L_{w}}{L_{s}}$ hash bits are pseudo-randomly permuted in the same way as in the encoding process, and the hash bits are divided into *L*_*w*_/4 subsets, as in the encoding process, calculating a modulo-2 sum of the four bits in each subset to obtain $64\times \frac{L_{w}}{L_{s}}$ “calculated check bits.”

These 64 calculated check bits are compared against the extracted check bits. Denote the number of extracted check bits in a segment by *N*_*E*_, and write *N*_*F*_ for the number of extracted check bits that are different from their corresponding calculated check bits, where *N*_*F*_ ≤ *N*_*E*_. If a segment has been tampered with, the probability that a calculated check bit is unequal to its corresponding extracted check bit is 0.5. Therefore, *N*_*F*_ follows a binomial distribution, and its probability distribution function is

PT,NF(l)=(NEl)×(0.5)NE×(0.5)NE-l,l=0,1,⋯,NE. (14)

**Fig 7:**
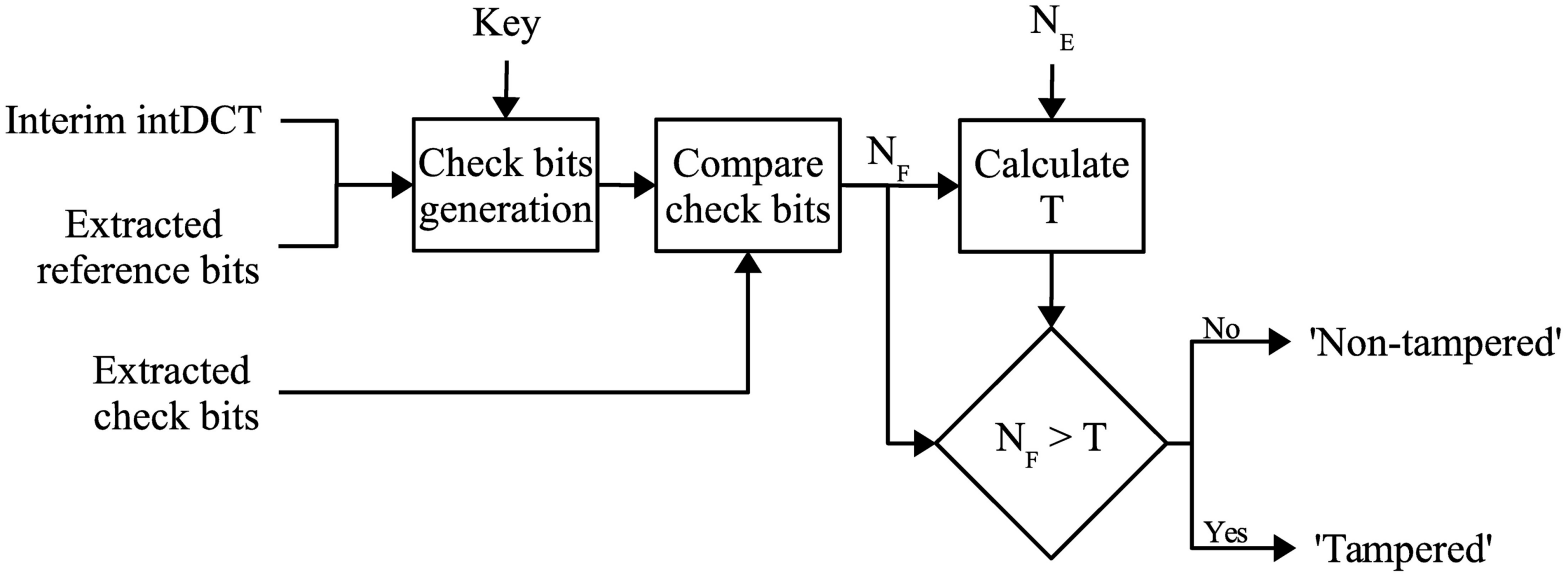
Block diagram for tamper identification.

For a given *N*_*E*_, an integer *T* is found such that

$$\sum_{l=0}^{T}P_{T},N_{F}\left(l\right)<10^{-9},$$ (15)

and

$$\sum_{l=0}^{T+1}P_{T},N_{F}\left(l\right)\geq 10^{-9}.$$ (16)

where *P*_*T*_, *N*_*F*_(*l*) is the probability distribution function of having *l* successes in *N*_*F*_ trials. If *N*_*F*_ > *T*, then the segment is regarded as “tampered,” but “non-tampered” otherwise. The probability of falsely identifying a tampered segment as a non-tampered one is less than 10^−9^. Fig 7 presents a block diagram with the steps for the tamper identification.

#### Signal restoration

In this final step, the original sample values from the “tampered” segments are restored. Mark the reference bits and sample values from each tampered segment as ‘NaN’ values to facilitate their differentiation from the reference bits and samples from the non-tampered segments in the next steps. The vectors and matrices from Eq 4 are recalculated with the extracted reference bits and the interim restored signal obtained so far (the time-domain signal obtained after watermark extraction). Because the received signal is quantized at 16 bits, each ‘NaN’ in the interim restored signal is converted to 16 ‘NaN’ values in the binary representation of the signal.

The 16 × *L*_*w*_ bits of the binary representation of the signal are divided into *Lw*/*n*_*g*_ groups as in the encoding process; each group contains *n*_*b*_ = *n*_*g*_ × 16 bits. The number of reliable reference bits in each bit-group, denoted by *n*_*t*_, may be less than the original *n*_*rb*_ reference bits from the encoding. Eq 4 implies that

$$\begin{array}{cc} \left[\begin{array}{c} r_{t}\left[s_{1}\right]\\ r_{t}\left[s_{2}\right]\\ \vdots \\ r_{t}\left[s_{n_{t}}\right] \end{array}\right] & =A_{t}^{\left(R\right)}\cdot [\begin{array}{c} b_{t}\left[1\right]\\ b_{t}\left[2\right]\\ \vdots \\ b_{t}\left[n_{b}\right] \end{array}],\\ t & =1,2,\cdots ,\frac{L_{w}}{n_{g}}. \end{array}$$ (17)

The **r**_**t**_ vectors contain the reliable extracted reference bits and $A_{t}^{\left(R\right)}$ is a matrix that has all the rows from *A*_*t*_ that correspond to the reliable extracted reference bits, *i.e*., all the rows in **r**_**t**_ with ‘NaN’ values are removed and the same rows from *A*_*t*_ are removed to obtain $A_{t}^{\left(R\right)}$. On the other side of Eq 17, the *n*_*b*_ bits in each bit-group contain two types of bits: 1) the missing bits from “tampered” segments, and 2) the recovered bits from other positions.

The assumption of this restoration strategy relies on the fact that if a small region of the signal was tampered with, then the number of missing bits in each **b**_**t**_ is small (because those missing bits are dispersed throughout different bit-groups) and do not affect the restoration.

In this way, the reliable reference bits and the non-missing bits in the **b**_**t**_ groups can provide enough information to recover the original values of the missing bits. Let **B**_**t**,**1**_ be a column vector that corresponds to the missing bits from **b**_**t**_, and **B**_**t**,**2**_ a column vector that corresponds to the recovered bits in **b**_**t**_, *i.e*., **B**_**t**,**1**_ is a column vector that corresponds to the rows in *b*_*t*_ that contain ‘NaN’ values and **B**_**t**,**2**_ is a column vector that corresponds to the rows in **b**_**t**_ with values different from ‘NaN’. Then Eq 17 can be reformulated as

$$[\begin{array}{c} r_{t}\left[s_{1}\right]\\ r_{t}\left[s_{2}\right]\\ \vdots \\ r_{t}\left[s_{n_{t}}\right] \end{array}]-A_{t}^{\left(R,2\right)}\cdot B_{t,2}=A_{t}^{\left(R,1\right)}\cdot B_{t,1},$$ (18)

where $A_{t}^{\left(R,1\right)}$ is a matrix constructed from the columns of $A_{t}^{\left(R\right)}$ that correspond to the missing bits in **b**_**t**_, and $A_{t}^{\left(R,2\right)}$ is a matrix constructed from the columns of $A_{t}^{\left(R\right)}$ that correspond to the recovered bits in **b**_**t**_. From Eq 18, the left side and the matrix $A_{t}^{\left(R,1\right)}$ are known variables, so only **B**_**t**,**1**_ has to be found. Let *n*_*mb*_ be the number of elements in **B**_**t**,**1**_. Then the size of the matrix $A_{t}^{\left(R,1\right)}$ is *n*_*t*_ × *n*_*mb*_. Then *n*_*mb*_ unknowns are solved for according to the *n*_*t*_ equations in the binary system, so the idea is to solve Eq 18 for **B**_**t**,**1**_, therefore obtaining the missing bits. With those missing bits, the 16-bit representation of the signal can be restored.

## Experimental results

To test this scheme, experiments with three CD-quality audio datasets were performed. These datasets are the Music Audio Benchmark (MAB) of the University of Dortmund [34], which has 1,886 musical excerpts with a duration of ten seconds at a sampling frequency of 44.1 KHz. The signals were originally in MP3 format, encoded at 128 kbps. These files were manually converted to waveform audio format (WAV) using the application Audacity [35], the sampling frequency of 44.1 KHz was maintained, and the quantization bits were set to 16 bits per sample. This dataset is divided into nine genres, namely, alternative, blues, electronic, folkcountry, funksoulrnb, jazz, pop, raphiphop, and rock. The Ballroom (Ball) dataset [36] has 698 musical excerpts with a duration of approximately 30 seconds at a sampling frequency of 44.1 KHz. The audio signals are monaural with a quantization of 16 bits per sample in WAV format. This dataset is divided into ten musical genres, namely, chachacha, jive, quickstep, rumba-americana, rumba-international, rumba-misc, samba, tango, viennese-waltz, and waltz. The third dataset was compiled by our research group (Ours) [37]. It was constructed for a quick test of the proposed scheme. This dataset contains 50 excerpts from music obtained from commercial CDs. The signals have a duration of 20 seconds in WAV format, at a sampling frequency of 44.1 KHz and a quantization of 16 bits per sample. It is divided into five genres, namely, jazz, orchestral, pop, rock, and vocal.

The scheme was evaluated in two phases. In the first phase, the perceptual impact of the encoding process on the watermarked signals was measured, to verify that its transparency is over -2 ODG. The second phase consisted of the evaluation of the restoration capability of the scheme after a content replacement attack with different percentages of severity had been applied to the watermarked signals. The restoration capability of the scheme is given by the ODG value between the host audio signals and the restored audio signals.

### Objective audio evaluation

The *objective difference grade* (ODG) is an objective measure to evaluate audio quality. The objective measurement algorithms model the listening behavior of humans: their output is a number that describes the audibility of the introduced distortions. The objective measurement method of the perceived audio quality (PEAQ) is an international standard, ITU-R BS.1387. This algorithm compares the difference between a reference signal (original) and a test signal (watermarked); both signals are processed by an auditory system that calculates an estimate of the audible components of the signal. These components can be considered as representations of the signals in the human auditory system. The internal representation is related to the masked threshold, which in turn is based on a psychoacoustic model. From these two internal representations, an audible difference is calculated and the cognitive model calculates the ODG value from the audible difference [38]. This ODG can take a value within the range from 0 to -4 and is defined as in Table 1.

**Table 1:** ODG values and their definition.

Impairment description	ODG
Imperceptible	0.0
Perceptible, but not annoying	-1.0
Slightly annoying	-2.0
Annoying	-3.0
Very annoying	-4.0

### Evaluation of the encoding process

Table 2 presents the mean (*μ*), standard deviation (*σ*), minimum, and maximum values measured with the peak signal-to-noise ratio (PSNR), and ODG between the host and watermarked audio signals. The inserted payload is the result of embedding the reference bits and check bits for each window of samples, and is approximately 24,800 bps (bits per second). In this table, the numbers in bold indicate mean ODG values over -1, which is one ODG point over the desired threshold. As can be seen from this table, the average ODG values for most of the genres in the datasets ‘MAB’ and ‘Ours’ are over -1, indicating that the difference between the host and watermarked audio signals is indistinguishable to a human listener. The average ODG values for all the audio signals in the three datasets are over -2 ODG, fulfilling the threshold required by the applications.

**Table 2:** Embedding* results for the tested datasets.

Dataset	Genre	PSNR				ODG			
		*μ*	*σ*	Min	Max	*μ*	*σ*	Min	Max
Ball	ChaChaCha	38.7	6.2	29.2	57.6	-1.1	0.3	-2.1	-0.4
	Jive	40.0	7.1	29.5	63.4	-1.1	0.3	-1.9	-0.5
	Quickstep	47.0	10.2	30.6	74.5	-1.0	0.4	-1.6	0.0
	Rumba	50.5	7.6	35.9	66.1	−0.9	0.3	-1.9	-0.4
	Tango	42.6	10.0	30.5	68.3	-1.4	0.4	-2.3	-0.6
	Waltz	48.5	11.9	35.7	72.9	-1.5	0.5	-2.2	-0.4
MAB	Alternative	50.6	10.9	28.0	74.5	−0.6	0.3	-1.7	0.1
	Blues	55.3	10.4	33.9	74.5	−0.5	0.3	-1.2	0.0
	Electronic	56.9	12.8	28.9	74.5	−0.6	0.4	-2.1	0.0
	Folkcountry	54.9	11.1	31.9	74.5	−0.6	0.3	-1.2	0.1
	Funksoulrnb	50.6	10.4	36.8	74.5	−0.6	0.3	-1.2	0.1
	Jazz	54.2	10.8	38.2	74.5	−0.7	0.3	-1.6	-0.2
	Pop	49.8	9.7	29.9	73.9	−0.6	0.3	-1.2	0.1
	Rock	50.7	11.7	34.1	74.5	−0.5	0.3	-1.2	0.1
Ours	Jazz	50.5	5.2	43.6	59.1	−0.8	0.2	-1.2	-0.5
	Orchestral	66.8	10.2	48.1	74.5	-1.3	0.5	-2.1	-0.7
	Pop	45.8	5.0	35.4	54.0	−0.9	0.2	-1.2	-0.7
	Rock	41.1	3.1	35.7	46.8	−0.9	0.1	-1.1	-0.7
	Vocal	51.8	4.4	44.6	58.4	−0.8	0.2	-1.4	-0.6

*Embedded payload = 24.8 kbps

#### Content replacement simulation

The second phase of evaluating the proposed scheme consisted in testing the restoration capability of the scheme after a content replacement attack had been applied to the watermarked audio signals. A content replacement attack in application scenarios like the ones already mentioned would carefully select samples that correspond to words in the audio signal and replace them with other words, silences, single tones, or sound effects. However, to evaluate a big set of watermarked audio signals, this process has to be automated. For this reason, the content replacement attack had to be simulated for this experimental setup. The simulated content replacement was performed following Algorithm 2, where |.| indicates the size of the signal, randi(.), min(.), and max(.) are functions that generate random integer numbers, and obtain the minimum and maximum values within a signal, respectively.

**Algorithm 2**: Simulation of a content replacement attack.

**Input**: Watermarked audio (*x*_wat_), frame size (fr_*s*_), percentage of attack (%_attck_)

**Output**: Attacked audio (*s*)

**1** num_samps_ ← ⌊fr_*s*_ × (%_attck_/100)⌋

**2** max_pos_ ← |*x*_wat_| − num_samps_

**3** rand_pos_ ← randi([1 max_pos_], 1, 1)

**4** *s* ← *x*_wat_

**5** *s*(rand_pos_: rand_pos_ + num_samps_ − 1) ← randi([min(*x*_wat_) max(*x*_wat_)], 1, num_samps_)

### Evaluation of the restoration process

The watermarked audio signals produced by the encoding process were attacked with the simulated content replacement attack described above. Three percentages for the attack were used: 0.1%, 0.2%, and 0.3%. The PSNR, MSE, and ODG values between the host and the restored signals were measured to determine the quality.

The mean (*μ*), standard deviation (*σ*), minimum, and maximum values of the PSNR, and MSE for the three datasets with a 0.1% attack, are presented in Table 3. As can be seen, with this percentage of attack, the scheme achieves perfect restoration in all of the genres of the three datasets, as indicated by the bold blue values in the minimum MSE column. An MSE value of 0 means that there is no error between the host and restored audio signals, *i.e*., perfect restoration has been obtained. It can also be seen that for the dataset ‘Ours’, perfect restoration is achieved for all the audio signals in all the genres, except for one audio signal in the ‘orchestral’ genre.

**Table 3:** PSNR and MSE results of restored signals attacked with 0.1%.

Dataset	Genre	PSNR				MSE			
		*μ*	*σ*	Min	Max	*μ*	*σ*	Min	Max
Ball	ChaChaCha	28.8	8.6	10.9	53.8	2.08 × 10^−3^	1.00 × 10^−2^	0.00	8.09 × 10^−2^
	Jive	28.6	11.2	7.1	51.7	4.47 × 10^−3^	2.58 × 10^−2^	0.00	1.93 × 10^−1^
	Quickstep	21.9	9.3	6.4	34.2	4.23 × 10^−3^	2.93 × 10^−2^	0.00	2.27 × 10^−1^
	Rumba	32.0	2.1	30.3	34.4	8.59 × 10^−5^	2.48 × 10^−4^	0.00	9.24 × 10^−4^
	Tango	28.5	14.3	8.3	53.7	7.37 × 10^−3^	2.78 × 10^−2^	0.00	1.47 × 10^−1^
	Waltz	28.1	13.1	16.4	53.3	1.08 × 10^−3^	4.01 × 10^−3^	0.00	2.31 × 10^−2^
MAB	Alternative	23.9	19.3	2.4	48.8	1.58 × 10^−2^	7.89 × 10^−2^	0.00	5.70 × 10^−1^
	Blues	21.3	1.1	20.1	22.2	2.64 × 10^−4^	1.43 × 10^−3^	0.00	9.76 × 10^−3^
	Electronic	18.9	11.2	3.1	29.1	6.09 × 10^−3^	5.37 × 10^−2^	0.00	4.89 × 10^−1^
	Folkcountry	28.9	22.4	6.2	51.0	2.86 × 10^−3^	2.62 × 10^−2^	0.00	2.42 × 10^−1^
	Funksoulrnb	26.8	3.3	22.3	29.3	2.41 × 10^−4^	9.69 × 10^−4^	0.00	5.87 × 10^−3^
	Jazz	34.0	11.3	27.4	47.0	8.20 × 10^−5^	3.78 × 10^−4^	0.00	1.82 × 10^−3^
	Pop	25.7	16.8	5.4	49.5	7.29 × 10^−3^	4.31 × 10^−2^	0.00	2.85 × 10^−1^
	Rock	27.0	14.1	15.2	51.2	1.13 × 10^−3^	4.93 × 10^−3^	0.00	3.03 × 10^−2^
Ours	Jazz	*PR	PR	PR	PR	0.00	0.00	0.00	0.00
	Orchestral	PR	PR	30.4	PR	9.02 × 10^−5^	2.85 × 10^−4^	0.00	9.02 × 10^−4^
	Pop	PR	PR	PR	PR	0.00	0.00	0.00	0.00
	Rock	PR	PR	PR	PR	0.00	0.00	0.00	0.00
	Vocal	PR	PR	PR	PR	0.00	0.00	0.00	0.00

*PR = Perfect Restoration

The mean (*μ*), standard deviation (*σ*), minimum, and maximum values of the ODG for the attacked and restored audio signals are presented in Table 4. From this table, it can be seen that the average ODG values for the attacked audio signals are close to -4 for all the genres in the datasets. A value of -4 ODG indicates very annoying distortion, which means that the attack is severe despite the percentage of the attack used. In this table, the mean ODG values in bold blue indicate values equivalent to perfect restoration (ODG ≥0). As can be seen, these ODG results are consistent with the PSNR and MSE results from Table 3, which demonstrate perfect restoration capabilities. From all the audio signals evaluated in the three datasets, perfect restoration is achieved for 87.3% of the signals, and with the remaining 12.7% signals, approximate restoration is achieved for signals attacked with 0.1%.

**Table 4:** ODG results of attacked and restored signals for 0.1% attack.

Dataset	Genre	Attacked				Restored			
		*μ*	*σ*	Min	Max	*μ*	*σ*	Min	Max
Ball	ChaChaCha	-2.8	0.6	-3.8	-1.3	-0.3	0.9	-3.6	0.2
	Jive	-2.7	0.6	-3.7	-1.6	-0.1	0.7	-3.1	0.2
	Quickstep	-3.1	0.6	-3.8	-1.3	0.0	0.6	-2.5	0.2
	Rumba	-3.4	0.4	-3.8	-1.9	0.1	0.2	-0.6	0.2
	Tango	-3.3	0.4	-3.8	-2.4	-0.3	0.9	-2.7	0.2
	Waltz	-3.5	0.3	-3.9	-2.7	0.0	0.5	-2.6	0.2
MAB	Alternative	-2.7	0.9	-3.9	-0.9	0.0	0.7	-3.8	0.2
	Blues	-3.0	0.6	-3.9	-1.4	0.1	0.5	-3.2	0.2
	Electronic	-3.0	0.8	-3.9	-1.0	0.1	0.6	-3.9	0.2
	Folkcountry	-3.0	0.7	-3.8	-1.3	0.2	0.3	-1.5	0.2
	Funksoulrnb	-2.7	0.8	-3.8	-0.7	0.0	0.7	-3.1	0.2
	Jazz	-3.1	0.7	-3.9	-1.1	0.1	0.4	-1.6	0.2
	Pop	-2.6	0.9	-3.8	-0.5	0.0	0.8	-3.8	0.2
	Rock	-2.2	0.8	-3.8	-0.7	0.0	0.7	-3.2	0.2
Ours	Jazz	-3.4	0.4	-3.8	-2.5	0.2	0.0	0.2	0.2
	Orchestral	-3.8	0.1	-3.9	-3.5	0.1	0.3	-0.8	0.2
	Pop	-3.6	0.2	-3.9	-3.4	0.2	0.0	0.2	0.2
	Rock	-2.1	0.5	-2.9	-1.5	0.2	0.0	0.2	0.2
	Vocal	-3.5	0.3	-3.9	-2.9	0.2	0.0	0.2	0.2

**Fig 8:**
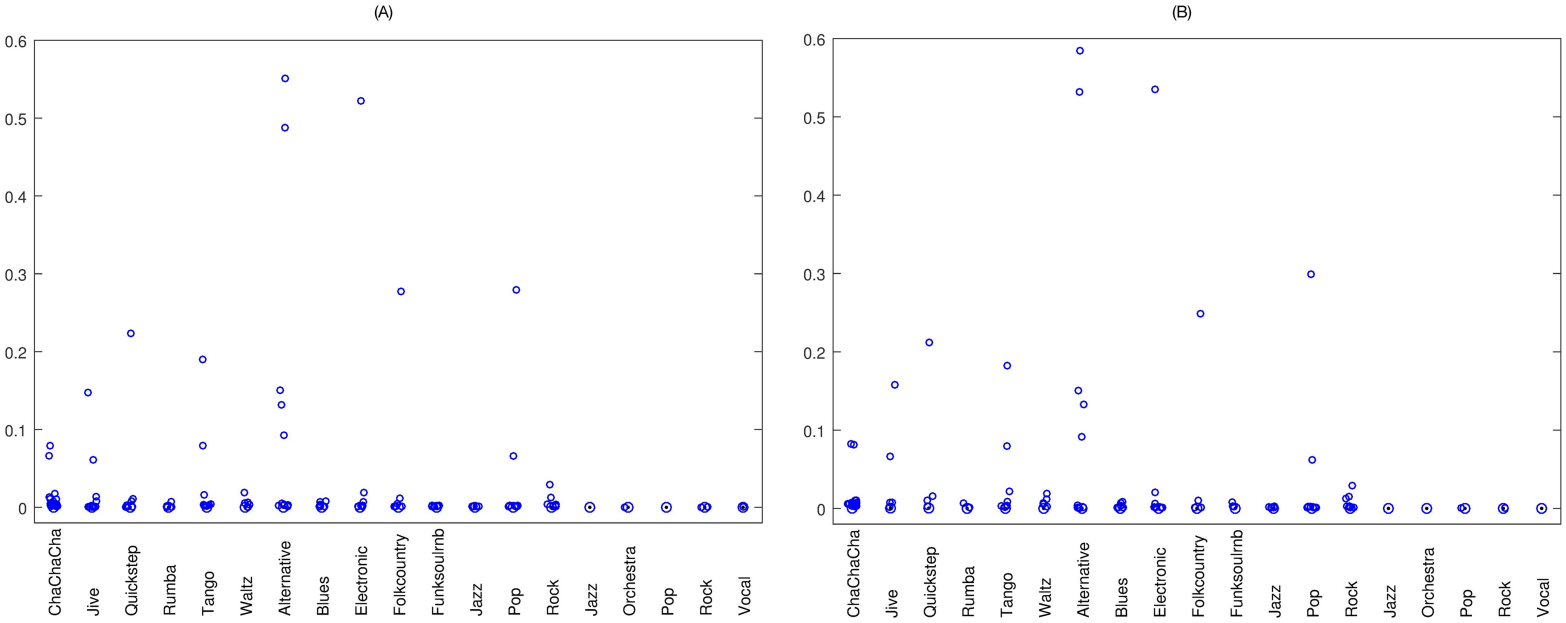
MSE results for three datasets. (A) 0.2% attack, (B) 0.3% attack.

**Fig 9:**
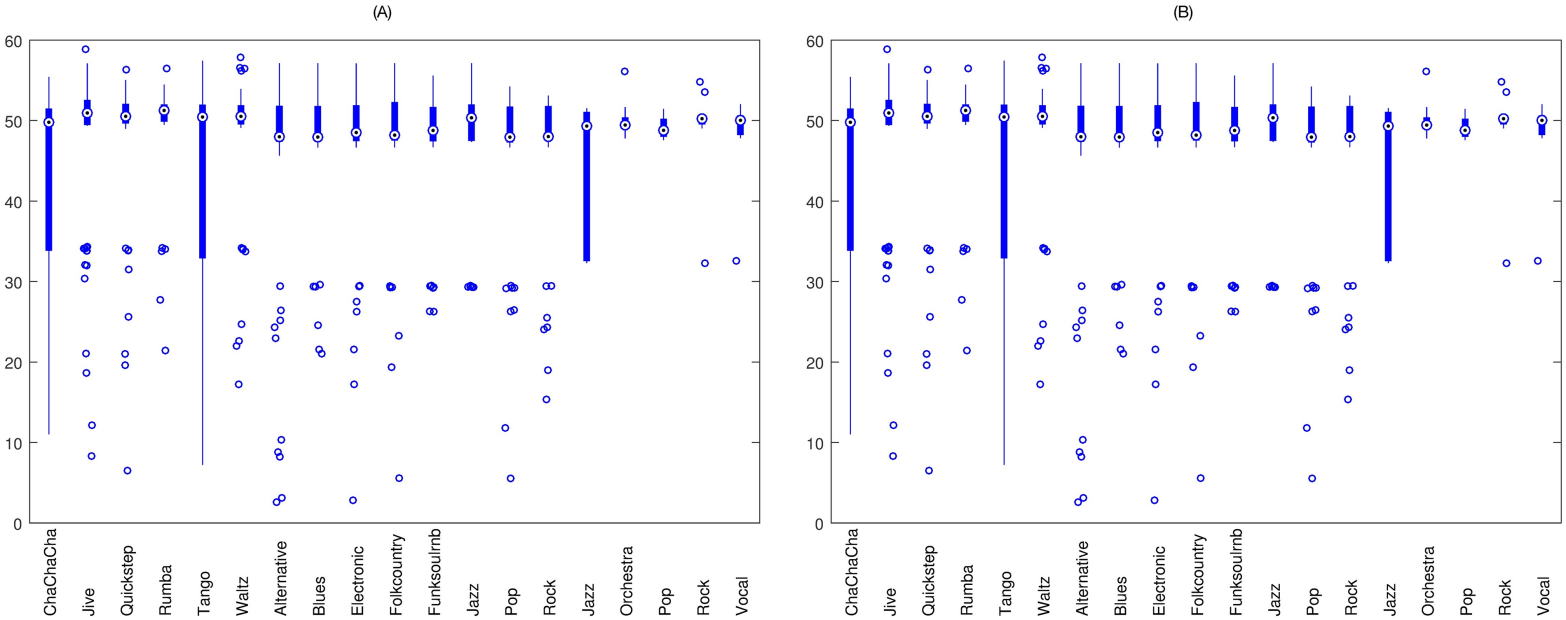
PSNR (dB) results for three datasets. (A) 0.2% attack, (B) 0.3% attack.

The distribution of MSE results for the restored audio signals of the three datasets with attacks of 0.2% and 0.3% are presented in Fig 8A and 8B, respectively. From these figures, it can be seen that most of the results are close to 0, which indicate small errors between the host and restored audio signals. The PSNR distribution for the results obtained from the restored audio signals of the three datasets, for attacks of 0.2% and 0.3%, are shown in Fig 9A and 9B, respectively. Here it can be seen that the standard deviation is greater than expected, which indicates that there are cases where the PSNR values are lower than 30 dB, for both the 0.2% and 0.3% attacks. However, for both these attacks, the restored PSNR values are over 30 dB for the great majority of the results, which indicates restoration with acceptable distortion.

The mean (*μ*), standard deviation (*σ*), minimum, and maximum values of the ODG for the attacked and restored audio signals for the 0.2%, and 0.3% attacks are presented in Table 5. As in Table 4, it can be observed that the average ODG values for the attacked audio signals are close to -4 for all the genres in the datasets, indicating annoying distortion in the attacked signals. The mean ODG values for the restored signals highlighted in bold blue indicate ODG values over -1. They indicate that the restored signals are very similar to the host ones, and the difference between host and restored is almost unnoticeable. As can be seen, most of the genres for the datasets ‘Ball’ and ‘Ours’ result in signals with high similitude to the host ones for both the 0.2% and 0.3% attacks. For the dataset ‘MAB’, it can be observed that the ODG results for all genres are over -2, which indicates perceptible but not annoying differences between the host and restored signals; this occurs for both the 0.2% and 0.3% attacks. The average ODG values in bold red indicate the cases where the ODG ≤ -2, and indicate that the differences between the host and restored signals are slightly annoying. This occurs for the ‘orchestral’ genre, where signals are low energy ones. The attacks are very noticeable because there is a great contrast between the random noise from the attack and the low energy samples from the rest of the signal. Although the scheme is capable of restoring certain samples in terms of their time domain values, these restored samples still have a noticeable contrast with the non-attacked low energy regions. Despite this, most of the results are over -2 ODG, and indicate that the quality of the restored audio signals is adequate for speech restoration and music distribution applications.

**Table 5:** ODG results of attacked and restored signals for 0.2% and 0.3% attacks.

%	Dataset	Genre	Attacked				Restored			
			*μ*	*σ*	Min	Max	*μ*	*σ*	Min	Max
0.2	Ball	ChaChaCha	-3.0	0.6	-3.7	-1.4	-1.0	0.7	-3.6	-0.2
		Jive	-2.9	0.6	-3.8	-1.5	−0.9	0.6	-3.6	-0.3
		Quickstep	-3.2	0.5	-3.8	-1.3	−0.9	0.5	-2.8	-0.4
		Rumba	-3.5	0.3	-3.9	-2.5	−0.8	0.4	-2.6	-0.4
		Tango	-3.4	0.4	-3.9	-2.4	-1.0	0.6	-2.5	-0.3
		Waltz	-3.5	0.3	-3.9	-2.6	−0.9	0.9	-3.7	-0.2
	MAB	Alternative	-2.9	0.8	-3.8	-1.1	-1.4	0.6	-3.8	-0.2
		Blues	-3.2	0.5	-3.8	-1.4	-1.6	0.6	-3.6	-0.5
		Electronic	-3.2	0.6	-3.9	-1.5	-1.7	0.9	-3.9	0.2
		Folkcountry	-3.1	0.6	-3.8	-1.4	-1.4	0.7	-3.4	0.2
		Funksoulrnb	-3.1	0.6	-3.9	-1.4	-1.4	0.6	-3.6	-0.3
		Jazz	-3.3	0.4	-3.9	-2.4	-1.6	0.9	-3.9	-0.6
		Pop	-3.0	0.7	-3.8	-1.1	-1.4	0.7	-3.9	0.2
		Rock	-2.8	0.6	-3.9	-1.4	-1.3	0.8	-3.8	-0.2
	Ours	Jazz	-3.5	0.3	-3.8	-2.6	−0.7	0.3	-1.1	-0.3
		Orchestral	-3.8	0.1	-3.9	-3.7	−2.4	1.3	-3.8	-0.7
		Pop	-3.7	0.1	-3.8	-3.5	−0.7	0.4	-1.4	-0.1
		Rock	-2.5	0.4	-3.2	-1.9	−0.5	0.1	-0.8	-0.2
		Vocal	-3.5	0.3	-3.8	-2.8	−0.7	0.3	-1.3	-0.2
0.3	Ball	ChaChaCha	-3.1	0.5	-3.7	-1.8	-1.0	0.7	-3.7	-0.2
		Jive	-3.1	0.5	-3.8	-1.6	−0.9	0.7	-3.5	-0.1
		Quickstep	-3.3	0.5	-3.8	-1.7	−0.9	0.5	-2.6	-0.1
		Rumba	-3.5	0.3	-3.9	-2.5	−0.9	0.5	-2.6	-0.3
		Tango	-3.4	0.3	-3.8	-2.5	-1.0	0.6	-2.5	-0.4
		Waltz	-3.5	0.3	-3.9	-2.7	-1.1	0.9	-3.6	-0.3
	MAB	Alternative	-3.0	0.6	-3.8	-1.0	-1.4	0.7	-3.8	-0.2
		Blues	-3.2	0.4	-3.8	-1.7	-1.7	0.6	-3.5	-0.5
		Electronic	-3.3	0.6	-3.9	-1.3	-1.8	0.9	-3.9	0.2
		Folkcountry	-3.2	0.6	-3.8	-1.5	-1.5	0.8	-3.5	0.2
		Funksoulrnb	-3.1	0.5	-3.9	-1.7	-1.3	0.8	-3.6	0.2
		Jazz	-3.4	0.4	-3.9	-1.9	-1.7	0.3	-3.8	0.2
		Pop	-3.0	0.6	-3.8	-1.2	-1.4	0.8	-3.8	0.2
		Rock	-2.8	0.6	-3.9	-1.6	-1.4	0.8	-3.8	0.2
	Ours	Jazz	-3.5	0.2	-3.8	-3.1	−0.4	0.4	-0.8	0.2
		Orchestral	-3.9	0.1	-3.9	-3.7	−2.0	1.6	-3.8	0.2
		Pop	-3.6	0.2	-3.9	-3.1	−0.7	0.3	-1.3	-0.3
		Rock	-2.5	0.5	-3.2	-1.9	−0.5	0.1	-0.6	-0.4
		Vocal	-3.6	0.3	-3.9	-3.1	−0.6	0.4	-1.1	0.2

Although the PSNR and ODG results for the 0.2% and 0.3% attacks might seem contradictory, what has occurred is the following. The PSNR results from Fig 9 are not as high as expected, because the restored samples are not as similar to the original samples in terms of their time-domain values, *i.e*., their numerical values are different. On the other hand, the ODG values from Table 5 indicate restoration with adequate distortion for the target applications. This means that, despite the fact that the numerical sample values are dissimilar, the perceived quality of the restored signals is adequate.

### Comparison to the related literature

In this subsection, a comparison with the literature related to our problem is given. The schemes [21, 22], and [23] are compared with our proposed scheme. In order to present a quantitative comparison for standardized transparency evaluation, implementations of all the schemes are needed, which is future research. Table 6 presents a comparison of the most significant properties of the schemes reported by the authors. As can be seen, the schemes of [21] and [22] present good transparency of their watermarked and restored signals. However, since they use a lossy compressed version of the signals to construct the information for self-recovery, these schemes cannot achieve perfect restoration. Furthermore, since [21] uses an LSB substitution in the time-domain representation of the signal to insert the payload, and since it is not encrypted prior to the insertion, the payload can be obtained by an attacker in a straightforward way, by just reading the LSB from each sample in the received signal. This seriously compromises the security of the scheme. Although [23] reports perfect restoration results, their experiments were carried out with only 100 signals selected by themselves, and not part of a standard repository; moreover, the reported results for the experiments with perfect restoration have SNR values under 10 dB, which is a bad audio quality. Our proposed scheme achieves high ODG values for both transparency and restoration quality of the signals, all experiments were carried out with signals from standard databases, and perfect restoration was also achieved.

**Table 6:** Comparison of self-recovery schemes for audio and speech signals.

Ref.	Domain	Payload (kbps)	Strategy	Tested signals	Transparency	Perfect restoration
[21]	Temporal	16	LSB substitution	2,750	Good	No
[22]	Temporal	NR*	Quantization	NR*	Good	No
[23]	Temporal	NR*	Difference expansion	100	Bad	Yes
Prop.	intDCT	24.8	Prediction error expansion	2,634	Very good	Yes

*NR = Not reported

## Discussion

The restoration capabilities of the proposed scheme, presented in the previous section, indicate that the scheme has effective restoration capabilities up to the tested 0.3% of content replacement. For the 0.2% and 0.3% attacks, the quality of the restored signals is adequate for the target applications. Furthermore, for the 0.1% attack, perfect restoration was achieved in 87.3% of the audio signals tested. From the results previously obtained, it can be appreciated that some applications would require a greater percentage of restoration than the current one; however, as far as we know, this is the first publication in the literature to propose a fully tested lossless audio restoration method. Lossless audio restoration is an open line of research, but with the presented results, a baseline has been provided, and future work will improve this scheme for its use in a wider range of applications.

Some strategies have to be further explored to increase the percentage of tampered samples that the scheme can restore or to obtain perfect restoration for 100% of the audio signals in the datasets. To improve the restoration capabilities, the payload of the scheme should be increased to allow the insertion of more reference bits. The proposed method is a solution to the problem of self-recovery for audio signals, which to the best of our knowledge did not exist in the literature. In addition to proposing a solution to the problem, the scheme satisfies the desired transparency threshold and provides effective restoration capabilities.

In the proposed self-recovery scheme, the problem of overflow that can occur when embedding the watermark is addressed in a pre-processing of the host audio signals. This pre-processing stage consists in adjusting the dynamic range of the signals to an integer representation. That adjusted dynamic range is then compressed to avoid overflow, in a similar fashion to that in [39].

Reversible watermarking techniques use more sophisticated strategies to deal with underflow and overflow, the most common one being the construction of a location map that indicates when these problems occur. However, the inclusion of a location map would require increasing the payload that is embedded into the signals. The increase of the payload caused by both restoration improvement and overflow solution could produce a payload size that cannot be embedded in the audio signals with the required transparency threshold. A strategy that does not require the construction of a location map has to be devised for the solution of underflow and overflow problems.

## Conclusions and future work

In this paper, a self-recovery scheme for audio signals has been introduced. The use of auditory masking properties was proposed for the selection of frequencies, and the mapping to the intDCT domain for the watermark embedding and extraction was also proposed. Because of the frequency selection in the intDCT domain, the scheme complies with an ODG threshold adequate for speech restoration or music distribution scenarios. The transparency requirement is one of the most challenging aspects of a self-recovery scheme, and as the experimental results demonstrate, the proposed strategy solves it.

Future efforts should improve the restoration capabilities of the scheme regarding the tolerance to attacks, as well as perfect restoration. The improvement can be achieved with the increase of payload capacity, to insert more reference bits. The inclusion of a synchronization strategy should also be considered to extend the solution for cropping and content replacement with duration change.

Content replacement attacks without duration change are being investigated, in part because of the applications where the scheme will be used, but also because this case is the basic model of the problem being addressed. Attacks such as cropping or content replacement with duration change are more general cases. From the solution of content replacement without duration change, the other attacks can be addressed by including a synchronization mechanism in the solution. The base case of content replacement is an open problem for practical applications. Future efforts will incorporate a synchronization strategy in the scheme for dealing with cropping and other forms of content replacement.

In this paper, a solution for audio self-recovery has been introduced. The strategy satisfies the transparency required by practical applications, which is one of the major difficulties when designing such schemes. In addition, the results demonstrate that the restoration capabilities of the scheme are effective.
